# Conflict-Driven Displacement and the Risk of Infectious Disease Outbreaks in the Middle East

**DOI:** 10.5334/aogh.5263

**Published:** 2026-06-16

**Authors:** Ilyas Abdullahi Khalif, Yusuf Abdullahi Hubow, Nour Ahmed Dahir, Narura Omar Mohamed

**Affiliations:** 1 Faculty of Health Sciences and Tropical Medicine, Somali National University, Mogadishu, Somalia; 2 Center for Health Research and Innovation, Somali National University, Mogadishu, Somalia

**Keywords:** conflict, displacement, infectious diseases, global health, Middle East

**To the Editor,**

Escalating geopolitical tensions, protracted conflicts shaped by historical and geopolitical forces, and recurrent regional military confrontations across the Middle East, including recent hostilities involving Iran, Israel, and allied actors, are driving large-scale displacement within and across borders. Millions of civilians are being uprooted into precarious internal displacement sites, informal settlements, and cross-border refugee movements, often within an already fragile regional health and political landscape. This regional crisis reflects a broader global trend in which forced displacement has increased sharply over the past decade, reaching historically unprecedented levels driven largely by armed conflict, political instability, and humanitarian crises, with major implications for infectious disease prevention and global health security. Conflict-driven displacement is a well-established catalyst for infectious disease emergence and transmission, primarily through forced mass movements, destruction of infrastructure, and disruption of basic services [1, 2]. Evidence from conflict settings in Syria, Gaza, and other fragile contexts shows that these dynamics not only deepen local humanitarian crises but also generate conditions that can rapidly ignite and amplify epidemics with potential cross-border implications [1–3].

Displaced populations in the region frequently reside in overcrowded shelters, refugee camps, and temporary settlements that fall far below minimum humanitarian standards for water, sanitation, and hygiene (WaSH). Internally displaced persons (IDPs) may experience particularly elevated infectious disease risks because they frequently remain trapped within active conflict zones where humanitarian access, healthcare, shelter, and protection systems are severely disrupted. In many settings, IDPs experience worse living conditions than refugees crossing international borders, who may have comparatively greater access to organized humanitarian assistance, formal camps, and health services. Studies from internally displaced populations in Gaza document extremely high incidences of acute respiratory infections (49.3%) and diarrheal disease (24.9%), reflecting the combined effects of overcrowding, inadequate shelter, unsafe water, poor sanitation, and disrupted healthcare access. Beyond generalized infectious disease vulnerability, recent conflicts in the Middle East have been associated with outbreaks or heightened risks of epidemic-prone diseases including cholera, measles, and poliomyelitis in fragile and displacement-affected settings, particularly where immunization programs, disease surveillance, and water systems have been disrupted. Acute respiratory infections spread rapidly in overcrowded shelters with poor ventilation, while diarrheal diseases are amplified by contamination of drinking water, inadequate sanitation infrastructure, and limited hygiene resources among displaced populations [4, 5]. Systematic reviews of conflict settings similarly identify population displacement, overcrowding, poor housing, and disrupted WaSH infrastructure as primary drivers of cholera, measles, acute diarrheal diseases, and other epidemic-prone infections after crises [1, 2]. Overstretched and under-resourced host communities in neighboring states experience additional pressure, with limited capacity to absorb displaced populations without compromising infection prevention and control [1, 2]. In such environments, infectious disease transmission is intensified not only by overcrowding and poor WaSH conditions but also by inadequate medical care, disrupted continuity of treatment, shortages of essential medicines, including antibiotics, and delayed access to diagnosis and clinical management. Overcrowded shelters with limited ventilation facilitate respiratory pathogen transmission, while untreated infections, interrupted antimicrobial access, and weakened infection prevention systems further increase morbidity and outbreak potential, particularly among children, malnourished individuals, older adults, and persons with chronic illness [2, 3, 6].

Concurrently, conflict undermines the very systems required to prevent, detect, and contain outbreaks. In several conflict-affected settings in the Middle East, damage to hospitals, ambulances, laboratories, water infrastructure, and public health systems, alongside interruptions to humanitarian access and insecurity affecting health workers, has severely constrained outbreak prevention and response capacity. Such disruptions weaken immunization, disease surveillance, infection prevention, laboratory confirmation, and continuity of essential care, thereby increasing vulnerability to epidemic-prone diseases among displaced populations. Armed violence routinely damages or destroys health facilities, reduces health workforce availability, and interrupts supply chains, leaving essential services such as immunization, maternal and child health, and emergency care only partially functional or completely suspended [1, 2, 6]. Disruption of disease surveillance systems, including early warning and laboratory confirmation, delays recognition of outbreaks and weakens timely response [2, 6, 7]. Evidence from fragile and conflict-affected states shows that inadequate preparedness, fragile health governance, and limited surveillance capacity magnify the impact of public health emergencies, with armed conflicts further curtailing disease control programs and routine vaccination [2, 6]. In this context, vaccine-preventable diseases such as polio and measles are at heightened risk of resurgence, as illustrated by recent outbreaks in conflict-affected territories dependent on emergency immunization campaigns under insecure conditions [3, 7].

From a global public health security perspective, conflict-driven displacement in the Middle East must therefore be recognized not only as a humanitarian catastrophe but as a major and escalating public health threat. Protecting displaced and host populations in this region is integral to preventing regional and global spread of infectious diseases. We argue that conflict-driven displacement should be reframed not only as a humanitarian emergency but also as a transboundary epidemic amplifier that links forced migration, health-system disruption, and regional health insecurity. Rather than relying solely on conventional humanitarian responses, outbreak prevention strategies should incorporate conflict-adapted public health mechanisms, including mobile vaccination teams for displaced populations, interoperable cross-border surveillance systems, rapid outbreak intelligence sharing, emergency WaSH packages in informal settlements, and protected humanitarian supply corridors for medicines, diagnostics, and laboratory services. This demands urgent, coordinated international action that combines humanitarian response, protection of civilian infrastructure, and sustained diplomatic efforts to reduce conflict-related disruption of health systems and essential public health services: robust humanitarian assistance to ensure safe WaSH services and adequate shelter; explicit protection of health facilities, humanitarian personnel, displaced civilians, and medical supply chains under international humanitarian law, alongside stronger accountability mechanisms to safeguard civilian and public health infrastructure during conflict; and investment in resilient, conflict-adapted disease surveillance and early warning systems that integrate camp, community, and cross-border data [1–3, 6, 7]. Strengthening routine and catch-up vaccination, supporting regional cooperation on outbreak preparedness, and embedding technological innovations such as digital surveillance and mobile health tools in conflict zones are critical to reducing epidemic risk [6, 7]. As tensions involving Iran, Israel, and neighboring states continue to destabilize the region, global health actors, donors, and political leaders must treat the prevention of infectious disease outbreaks among displaced populations as a core pillar of diplomacy, peacebuilding, and global health security rather than a secondary humanitarian afterthought.

## Author Access Statement

All authors had access to the information used in preparing this manuscript and participated in writing and reviewing the manuscript.
